# c-Fos Expression during Temporal Order Judgment in Mice

**DOI:** 10.1371/journal.pone.0010483

**Published:** 2010-05-05

**Authors:** Makoto Wada, Noriyuki Higo, Shunjiro Moizumi, Shigeru Kitazawa

**Affiliations:** 1 Department of Physiology, Juntendo University School of Medicine, Tokyo, Japan; 2 CREST, Japan Science and Technology Agency, Tokyo, Japan; 3 Neuroscience Research Institute, National Institute of Advanced Industrial Science and Technology (AIST), Tsukuba, Japan; 4 Cognitive Functions Section, Department of Rehabilitation for Sensory Functions, Research Institute of National Rehabilitation Center for Persons with Disabilities, Tokorozawa, Saitama, Japan; Kyushu University, Japan

## Abstract

The neuronal mechanisms for ordering sensory signals in time still need to be clarified despite a long history of research. To address this issue, we recently developed a behavioral task of temporal order judgment in mice. In the present study, we examined the expression of c-Fos, a marker of neural activation, in mice just after they carried out the temporal order judgment task. The expression of c-Fos was examined in C57BL/6N mice (male, n = 5) that were trained to judge the order of two air-puff stimuli delivered bilaterally to the right and left whiskers with stimulation intervals of 50–750 ms. The mice were rewarded with a food pellet when they responded by orienting their head toward the first stimulus (n = 2) or toward the second stimulus (n = 3) after a visual “go” signal. c-Fos-stained cell densities of these mice (test group) were compared with those of two control groups in coronal brain sections prepared at bregma −2, −1, 0, +1, and +2 mm by applying statistical parametric mapping to the c-Fos immuno-stained sections. The expression of c-Fos was significantly higher in the test group than in the other groups in the bilateral barrel fields of the primary somatosensory cortex, the left secondary somatosensory cortex, the dorsal part of the right secondary auditory cortex. Laminar analyses in the primary somatosensory cortex revealed that c-Fos expression in the test group was most evident in layers II and III, where callosal fibers project. The results suggest that temporal order judgment involves processing bilateral somatosensory signals through the supragranular layers of the primary sensory cortex and in the multimodal sensory areas, including marginal zone between the primary somatosensory cortex and the secondary sensory cortex.

## Introduction

How the brain orders successive events in time has become a subject of intense investigation in recent years [1], [2], [3], [4], [5], [6], [7], [8], [9], [10], [11], [12] after a long history of research in this field [13], [14], [15], [16], [17], [18], [19]. However, the neuronal mechanisms for ordering sensory signals in time still need to be clarified. To address this issue, we recently developed a behavioral task of temporal order judgment in mice [20]. In this task, a mouse is required to orient its head toward the first or second of two air-puff stimuli that are delivered to the right and left whiskers at relatively small stimulus onset asynchronies from 50 to 750 ms.

In this study, we examined the expression of c-Fos in mice just after they carried out temporal order judgments of whisker stimulations. c-Fos is one of the immediate early genes that are induced by calcium influxes resulting from cell excitation[21] and thus is considered to be a marker of task-related neural activation [22], [23], [24], [25], [26], [27], [28], [29], [30].

In previous studies with whisker stimulations in rats [23], [26], c-Fos expression was most evident in the granular layer (layer IV) of the primary somatosensory cortex, which receives direct projections from the ventral posteromedial thalamic nucleus (VPM). To discriminate these non-task-specific activations from those that are critical for ordering somatosensory signals, we prepared a control group that received two successive stimuli to unilateral (right or left) whiskers in each trial and were required to orient to the side of the successive stimuli. The control group thus received as many stimuli and made as many responses as the test group, but had no chance to order the bilateral stimuli. We compared c-Fos expression patterns in the test and control groups and found significantly higher c-Fos expression in several areas of the brain, including the superficial layers of the barrel cortex and secondary sensory cortices, in the test mice.

## Methods

### Subjects

Fifteen male mice (C57BL6NCrj) were used. They were assigned to three groups (5 mice for each): one test group that performed temporal order judgment and two control groups. The mice included in the test group were those that participated in our previous study [20]. Their body weights ranged from 20 to 25 g at the beginning of behavioral training, and the mice were maintained at greater than 90% of their ordinary body weights with free feeding throughout the training period. The mice received training sessions of 30–60 min each weekday. All experimental protocols were approved by the Ethics Review Committee for Animal Experimentation of Juntendo University School of Medicine and followed the Guiding Principles for the Care and Use of Animals approved by the Council of the Physiological Society of Japan.

### Apparatus and task procedures

**Figure 1:**
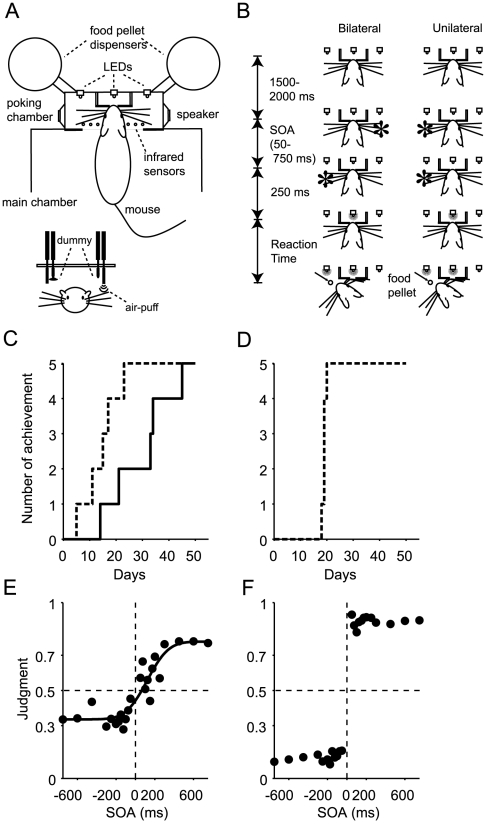
Apparatus (A), task procedures (B), and task performances (C–F). (A) Overhead and front views of the apparatus. Two pairs of tubes were placed vertically within the nose-poking chamber to deliver successive puffs of air (stimuli) to the whiskers. One tube (dummy) of each pair delivered air-puffs outside the chamber. (B) Examples of bilateral stimuli (first stimulus delivered from the right, second from left) and unilateral stimuli (both stimuli delivered from the left) are illustrated. The mouse in the figure would be rewarded if it oriented its head toward the side where the second stimulus had been delivered (bottom panels) following the illumination of the light-emitting diode (LED) in the center of the array of three LEDs. SOA, stimulus onset asynchrony. (C, D) Cumulative ratio of task achievement in unilateral (broken lines) and bilateral (solid lines) trials. The data in C and D are for the test group (n = 5) and the unilateral group (n = 5), respectively. (E) Performance of temporal order judgment in the test group. The order-judgment probability that the right-side whiskers were stimulated earlier than the left-side whiskers is plotted against the stimulus onset asynchrony (SOA). A positive SOA indicates that the right-side whiskers were stimulated first. The response was fitted with a sigmoid function (r^2^ = 0.86) with asymptotes of 0.34 and 0.78, a temporal resolution of 173 ms, and a horizontal bias of 121 ms. (F) Performance of spatial orienting in the unilateral group. The probability of rightward orienting is plotted against the SOA. Positive and negative SOA values indicate that stimuli were delivered to the right (right-right) and the left (left-left), respectively. (A), (B), (C) and (E) were derived from Fig. 1A, C, Fig. 3A and Fig. 6A of Wada et al.[20], respectively.

Mice were trained and tested in an operant box (Fig. 1A) that was designed specifically for our study (O'Hara & Co., Tokyo, Japan) as described elsewhere [20]. Briefly, the box consisted of a large main chamber and a smaller nose-poking chamber (Fig. 1A). In the nose-poking chamber, there was a small, round hole in the center (5 mm in diameter) into which the mouse poked its nose. Two pairs of tubes were placed vertically within the nose-poking chamber to deliver a puff of air (the stimulus) to the long whiskers.

An array of infrared photosensors (n = 11, 4-mm-intervals) was placed at the border between the main and the nose-poking chamber to detect nose poking and head orientation. Two food pellet dispensers were attached to the right and left side of the nose-poking chamber. Three yellow light-emitting diodes (LEDs) were attached to the wall in front of the head of the animal (positioned to the left, center, and right of the head) to illuminate the food pellet or to deliver the go signal for a response.

The box was isolated within a sound-attenuating, light-proof box (Music Cabin Co., Kawasaki, Japan). All experiments were carried out in the dark, except when the yellow LEDs were illuminated. During experiments, the box was filled with white noise (85 dB) from two speakers placed within the nose-poking chamber to mask the sound of the delivery of the air puffs.

After the mouse poked its nose into the small hole (top panels in Fig. 1B), two successive stimuli were delivered bilaterally (Bilateral) or unilaterally (Unilateral). Examples of bilateral stimuli (first stimulus delivered from the right, second from left) and unilateral stimuli (both stimuli delivered from the left) are illustrated in Fig. 1B. The mouse in the figure would be rewarded if it oriented its head toward the side where the second stimulus had been delivered (bottom panels) following the illumination of the light-emitting diode (LED) in the center of the array of three LEDs. It is worth noting that not only right-then-left but also left-then-right stimuli were delivered in a randomized order as bilateral stimuli, and not only left-then-left but also right-then-right stimuli were delivered as unilateral stimuli. Five mice (test group) received both types of stimuli (right-left, left-right, right-right and left-left stimuli), whereas five other mice (unilateral group) received only unilateral stimuli (both right-right and left-left stimuli). We prepared another group that did not receive any stimuli (home-cage group, n = 5).

### Behavioral training

Each mice in the test group (n = 5) was required to orient its head toward the first (n = 2) or second (n = 3) of two air-puff stimuli that were delivered bilaterally to the right and left whiskers (bilateral stimuli, Fig. 1B) or unilaterally to the right (right-right) or the left (left-left) whiskers (unilateral stimuli, Fig. 1B). The criterion of task achievement was set at 70% correct responses for three consecutive days. Details of the training procedures can be found elsewhere [20]. During the final week prior to being sacrificed, the mice received only bilateral air-puff stimuli with various stimulus onset asynchronies (SOAs) from 50 to 750 ms. One of the two control groups was a unilateral group that received unilateral but not bilateral stimuli; they were required to orient to the side of successive stimuli (spatial orienting) but never to judge the order of the two stimuli. Before histological analysis, these mice received unilateral air-puff stimuli with various SOAs from 50 to 750 ms. The other control group was a home-cage group that experienced the same food restriction schedule and spent 30–60 min/day in the same soundproof box where the tasks of the other groups were conducted. All of the groups experienced 5 to 9 months of tasks and/or scheduled food restriction.

Three of the five mice in the test group were tested further to determine whether they responded to stimuli delivered to the whiskers in particular [20]. For this purpose, the long whiskers on both sides of the snout were removed close to the surface of the skin using scissors, under diethyl ether anesthesia. In subsequent three sessions, we confirmed that the responses of whiskerless mice to bilateral stimuli (SOA = 710–750 ms) dropped near to the chance level [20]. The three whiskerless mice were trained again for two months after the whiskers had regrown in two weeks. Due to the whisker removal test and re-training after the regrowth, the median age at sacrifice was older in the test group (8 months) than those in the unilateral group (5 months) and the home-cage group (6 months). The difference between the test group and the unilateral group was significant (p = 0.048, Wilcoxon rank-sum test).

### Histology

The last session was continued up to the time when the mice ceased nose-poking for 5 min in the test and unilateral groups (28 to 157 trials during 30 to 144 min for each mouse). Five min after the last trial, the mice were immediately anesthetized by diethyl ether, sacrificed with an overdose of pentobarbital, and perfused intracardially with 60 ml of 0.01 M phosphate-buffered saline (PBS) followed by 30 ml of 4% paraformaldehyde in 0.01 M PBS. The brains were removed and stored overnight in 4% paraformaldehyde at 4°C. The next morning, the brains were first transferred to 15% sucrose and then to 30% sucrose at 4°C until they sank. The brain blocks were mounted in O.C.T compound (Miles Inc., Elkhart, IN), rapidly frozen in a dry ice/acetone bath, and then stored at −65°C until dissection. Coronal brain sections (18 µm) were cut on a freezing microtome (Cryocut 3000, Leica, Nussloch, Germany). The sections were mounted on pre-coated slides (MAS-coated slide S9441, Matsunami Glass Ind., Ltd., Osaka, Japan) and then processed for immunostaining.

**Figure 2:**
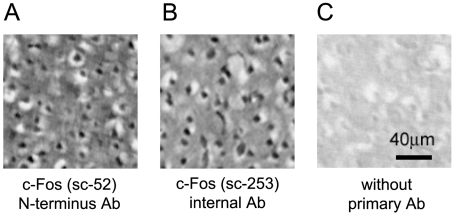
Specificity of the anti-c-Fos antibodies. Immunostaining of the primary somatosensory cortex with an N-terminus antibody, SC-52 (A), and an internal epitope antibody, SC-253 (B), is shown with a control of staining without primary antibody (C). The samples were prepared from neighboring sections in one subject.

For single-labeling of cells that expressed c-Fos protein, the avidin-biotin peroxidase method was used with a polyclonal rabbit antibody specific for c-Fos (SC-52; Santa Cruz Biotechnology, Santa Cruz, CA). The sections were first treated with 0.01% H_2_O_2_ in methanol for 20 min to destroy endogenous peroxidases and then incubated for 1 h in 0.1% Triton-X 100 and 1.5% normal goat serum to minimize nonspecific labeling. Next, the tissue sections were incubated for 72 h at 4°C in a 1∶4000 dilution of anti-c-Fos antibody (SC-52 in 0.01M PBS with 0.5% bovine serum albumin). The sections were washed, placed for 1.5 h in a 1∶200 dilution of biotinylated goat anti-rabbit antibody (Vectastain, ABC Elite kit, Vector Laboratories, Burlingame, CA), washed again, and then placed for 1 h in a 1∶200 dilution of avidin–biotin complex (Vectastain, ABC Elite kit). The peroxidase activity was visualized with a nickel-enhanced coloring solution (0.2 mg/ml diaminobenzidine: DAB, 0.02% H_2_O_2_, 0.03% nickel ammonium in Tris-buffered saline). After washing with distilled water, the sections were dehydrated through a series of 70, 90, 99, and 100% ethanol (1 min at each concentration), transferred to xylene for three washes of 1 min each, and then covered with coverslips and mounting medium (MP-500, Matsunami Glass Ind., Ltd., Osaka, Japan). Figure 2A shows cell nuclei stained with the c-Fos antibody (SC-52, N-terminus epitope). The specificity of the antibody (SC-52) had been previously confirmed by Western blot analysis (data sheet from Santa Cruz Biotechnology). We additionally confirmed in neighboring sections that another anti-c-Fos antibody that combines with an internal epitope (SC-253; 1∶4000, Santa Cruz Biotechnology) yielded similar results (Fig. 2B). We further confirmed that there was no nonspecific labeling in the absence of the primary c-Fos antibody (Fig. 2C).

Immunohistofluorescence was used to double-label cells that expressed c-Fos and one of the following: glial fibrillary acidic protein (GFAP), gamma-aminobutyric acid (GABA), alpha-subunit of type II calcium-calmodulin-dependent protein kinase (CAMKIIα), and neuronal nuclei (NeuN). The primary antibodies to GFAP (rabbit polyclonal antibody Z0334, 1∶500; Dako, High Wycombe, UK), GABA (guinea pig polyclonal antibody AB175, 1∶1000; Chemicon, Temecula, CA), and c-Fos (rabbit polyclonal antibody SC-52 or goat polyclonal antibody SC-52G, 1∶1000) were visualized with Alexa Fluor 488 and Alexa Fluor 594 fluorophore-labeled secondary antibodies (Invitrogen, Carlsbad, CA). The sections were incubated for 1 h in 0.1% Triton-X 100 and 1.5% normal goat or horse serum to minimize nonspecific labeling. The tissue sections were washed and then incubated overnight at 4°C in 0.5% BSA and PBS containing anti-c-Fos antibody and the other antibody. Then the sections were washed and placed for 2 h in a 1∶1000 dilution mixture of fluorophore-labeled antibodies. When the fluorophore-labeled secondary antibodies consisted of a goat anti-rabbit antibody and an anti-goat antibody, the anti-goat secondary antibody was reacted first.

The primary biotinylated anti-NeuN antibody (mouse monoclonal antibody MAB377B, 1∶100; Chemicon, Temecula, CA, USA) was visualized with Alexa Fluor 594 streptavidin (Invitrogen, Carlsbad, CA, USA), while the anti-c-Fos antibody was visualized with Alexa Fluor 488 fluorophore-labeled secondary antibody (Invitrogen). The sections were incubated for 1 h in 3.6% MOM blocking reagent (Mouse on Mouse immunodetection kit; Vector Laboratories). The tissue sections were washed, incubated for 5 min in MOM diluents (Mouse on Mouse immunodetection kit; Vector Laboratories), and then incubated overnight at 4°C in MOM diluents containing the anti-c-Fos antibody and the biotinylated anti-NeuN antibody. Next, the sections were washed and placed for 1 h in 1∶500 dilution mixtures of fluorophore-labeled antibodies and the streptavidin.

The primary anti-CAMKIIα antibody (mouse monoclonal antibody MAB3119, 1∶50; Chemicon, Temecula, CA) was visualized with a biotinylated anti-mouse antibody (Mouse on Mouse immunodetection kit; Vector Laboratories) and Alexa Fluor 594 streptavidin (Invitrogen), while the anti-c-Fos antibody was visualized with Alexa Fluor 488 fluorophore-labeled secondary antibody (Invitrogen). The sections were incubated for 1 h in 3.6% MOM blocking reagent (Mouse on Mouse immunodetection kit; Vector Laboratories). The tissue sections were washed, incubated 5 min in MOM diluents, and then incubated overnight at 4°C in MOM diluents containing anti-c-Fos antibody. Next, the sections were washed and placed for 1 h in a 1∶500 dilution of the Alexa Fluor 488 fluorophore-labeled antibody for the c-Fos antibody. After acquiring the images for c-Fos staining, the sections were washed again and incubated for 5 min at 90°C in 0.01 M citric acid (pH 6.0) for antigen retrieval. After washing, the sections were incubated for 1 h in 3.6% MOM blocking reagent (Mouse on Mouse immunodetection kit; Vector Laboratories). The tissue sections were washed again, incubated for 5 min in MOM diluents (Mouse on Mouse immunodetection kit; Vector Laboratories), and then incubated overnight at 4°C in MOM diluents containing the anti-CAMKIIα antibody. The sections were washed, placed for 10 min in a 1∶250 dilution of biotinylated anti-mouse antibody (Mouse on Mouse immunodetection kit; Vector Laboratories), washed again, and placed for 5 min in a 1∶200 dilution of Alexa Fluor 594 streptavidin. These sections were finally washed and covered with coverslips and mounting medium (Vectashield H-1400; Vector Laboratories).

### Analysis of single c-Fos-labeled sections

The level of each coronal section from the bregma was determined according to a mouse brain atlas[31]. Tiled images of each c-Fos stained section were captured with a microscope (BX-60, Olympus, Tokyo, Japan; 4X or 10X) and a 3-CCD color video camera (DXC-950, Sony, Tokyo, Japan); a composite of the images was then constructed for each section by an image analyzer (MCID; Imaging Research Inc., Ontario, Canada). The details are as described in Higo et al. [32], [33], [34]. For each mouse, two to four neighboring sections were captured at each of the five levels (+2, +1, 0, −1, and −2±0.2 mm from the bregma). We took every other section for capture so that these neighboring sections were at least 18 µm apart from each other.

#### Positive Cell Density Map (PCDM)

**Figure 3:**
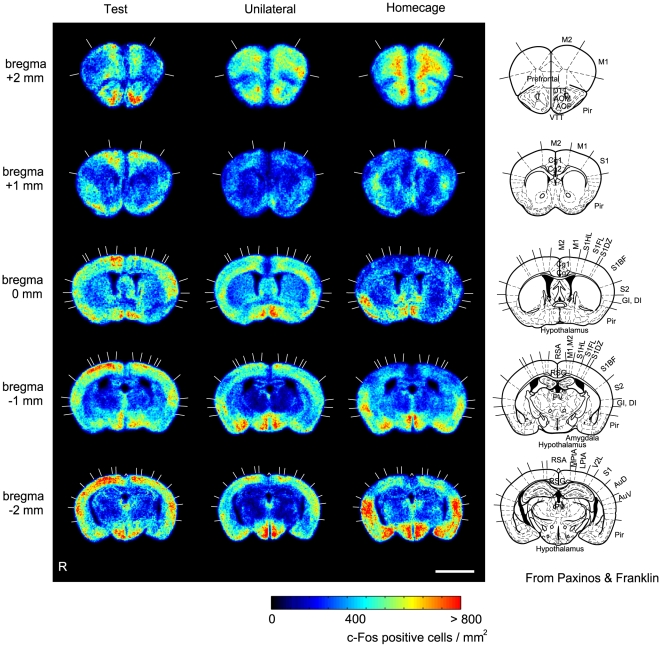
c-Fos-positive cell density maps (PCDMs) in three task groups. PCDMs at five different levels (bregma +2, +1, 0, −1, and −2 mm) are shown in rows for each group (columns). In the rightmost column, figures from the brain atlas (Paxinos & Franklin, 2001) are shown for comparison. Abbreviations: M1, primary motor cortex; M2, secondary motor cortex; Pir, piriform cortex; Tu, olfactory tubercle; AOP, anterior olfactory nucleus, posterior; AOM, anterior olfactory nucleus, medial; Cgl, cingulate cortex, area 1; Cg2, cingulate cortex, area 2; S1, primary somatosensory cortex; S1BF, primary somatosensory cortex, barrel field; PV, paraventricular hypothalamic nucleus; RSA, retrosplenial agranular cortex; RSG, retrosplenial granular cortex; MtPA, medial parietal association cortex; LtPA, lateral parietal association cortex; AuD, secondary auditory cortex, dorsal; AuV, secondary auditory cortex, ventral. Scale bar: 3 mm.

The composite image was FFT band-pass filtered with an NIH image-J program (NIH, Bethesda, MD) to eliminate low-frequency drifts (>20 pixels = 50 µm) and high frequency noises (<1 pixel = 2.5 µm). The filtered image was further analyzed with a homemade program that was developed on Matlab with image prorocessing toolbox (Mathworks Inc., Natick, MA). A c-Fos positive cell density map was prepared for each section by automatically detecting c-Fos positive cells and by counting the number of immunostained cells at each 100 µm×100 µm square compartment. For each mouse, we created a positive cell density map (PCDM) at each of the five levels from the bregma (+2, +1, 0, −1, and −2±0.2 mm). Finally, the PCDMs were normalized to a standard section and averaged for each group of five mice at each of the five levels (Fig. 3). Details of methodological considerations were as described previously [35], [36].

#### Statistical parameter mapping of immunopositive cell density

**Figure 4:**
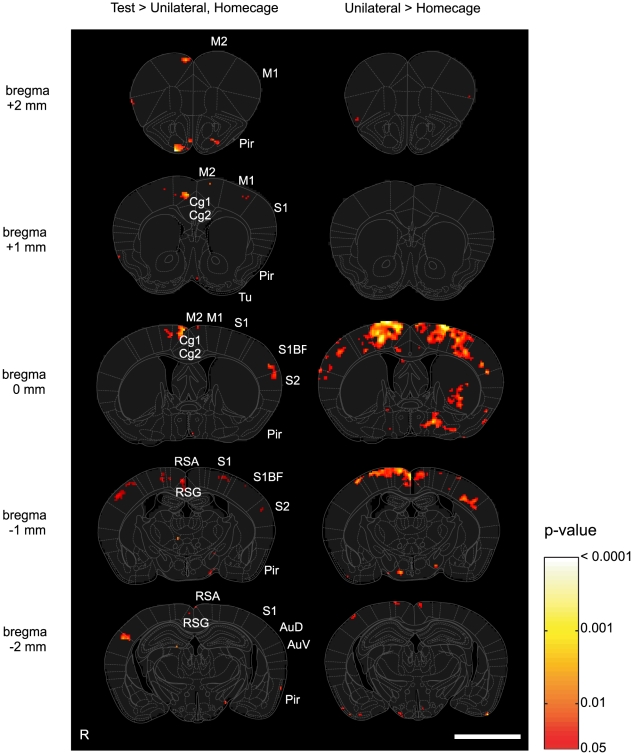
c-Fos statistical parametric mapping. (A) Areas that showed significantly greater c-Fos-positive cell densities in the test group than those in the unilateral group (p<0.05, uncorrected) masked by test vs. home-cage contrast (p<0.05, uncorrected). Block-by-block t-tests were applied between two groups. (B) Areas that showed significantly greater c-Fos-positive cell densities in the unilateral group than those in the home-cage group. The abbreviations are the same as in Fig. 3. Scale bar: 3 mm.

In a manner similar to our previous study [35], we carried out a block-by-block between-group comparison of c-Fos-positive cell densities. T-tests were repeatedly applied to each block, after spatially smoothing each PCDM with a Gaussian filter of the block size (S.D. = 100 µm). We first examined whether the test group (n = 5) showed a greater c-Fos cell density than the home-cage group (n = 5). In blocks where the p-value was less than 0.05 (uncorrected), we further examined whether the test group (n = 5) showed a greater c-Fos cell density than the unilateral group (n = 5). The p-value of the t-test at each block (<0.05, uncorrected) was mapped to show areas that would be contributing to the temporal order judgment (Fig. 4, left panel). We also compared the unilateral group with the home-cage group to show areas that responded to tactile stimuli and head-orienting movements required for responses (Fig. 4, right panel).

#### Laminar analysis in the barrel cortex

**Figure 5:**
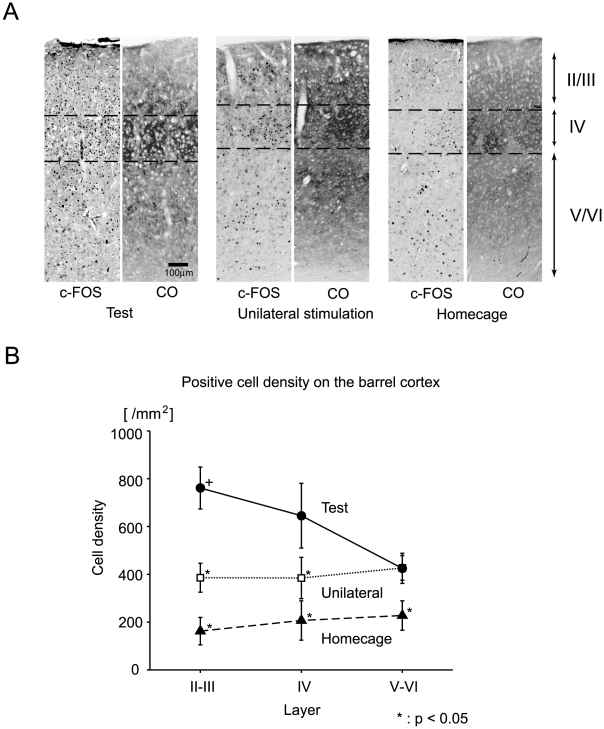
Laminar analyses in the barrel field of the primary somatosensory cortex. (A) Representative images of c-Fos immunostaining (c-Fos) and cytochrome oxidase staining (CO) are shown for each of the three groups. (B) c-Fos-positive cell density (ordinate) plotted against layers in the barrel cortex (abscissa). Different symbols show different groups. Error bars show the standard error of the mean. Data were obtained from both hemispheres for each subject. Thus, each symbol represents the mean of ten data points from five subjects of each group. Asterisks show that the mean values were significantly smaller than that of the supragranular c-Fos-positive cell densities of the test group (cross) after a Bonferroni correction for multiple comparisons (α = 0.05).

The number of positive cells was counted for different layers in the bilateral barrel field of the primary somatosensory cortex (bregma −0.8 to −1.2 mm). The boundaries between the layers were determined by cytochrome oxidase staining of neighboring sections (Fig. 5A, CO). Stained cells were counted by MCID (Imaging Research Inc.) in 600-µm-wide vertical strips across layers II-III, IV, and V-VI. The c-Fos-positive cells were counted in two to four neighboring sections for each mouse, and the mean positive cell density for each layer was calculated and assigned to each subject. The mean values among the task groups for each layer were compared using the Bonferroni correction for multiple comparisons.

As a control, we also measured c-Fos-positive cell densities in the paraventricular hypothalamic nucleus (bregma −0.8 to −1.2 mm, 250 µm×250 µm squares).

## Results

### Behavioral data

Five mice were included in the test group and were derived from the seven that achieved a learning criterion for both unilateral and bilateral stimulation tasks in our previous study [20]. The five mice achieved the criterion by 21 days for the unilateral stimulation task (broken line in Fig. 1C) and by 46 days for the bilateral stimulation task (solid line in Fig. 1C). A psychometric function of the five mice (Fig. 1E) showed that the mice correctly judged the order of bilateral whisker stimulations in about 70% of the trials when the stimulation interval was longer than 200–300 ms. It is worth noting that the sigmoid function (r^2^ = 0.86) had a horizontal bias of 121 ms, showing that the mice judged two stimuli as simultaneous when the right whiskers were stimulated earlier by 121 ms. The five mice participated in only the bilateral stimulation task (temporal order judgment) on the day of sacrifice and within 1 week prior to the sacrifice. They performed 29–65 trials in 37–109 min in the last experiments. They were rewarded in 66–84% (78±7%, mean ± S.D.) of the trials because they were rewarded in all trials with stimulation onset asynchronies of less than 200 ms irrespective of whether they responded correctly.

The five mice in the unilateral stimulation group achieved the learning criterion for the unilateral stimulation task by 20 days from the onset of training (Fig. 1D). The mice received as many air-puff stimuli (two) per trial as those in the bilateral stimulation group, but the stimuli were always delivered unilaterally to the right or the left whiskers (right-right or left-left stimuli). They were always required to orient to the side of stimulation and never had a chance to order bilateral stimuli. They responded correctly in about 90% of trials irrespective of the interval between successive stimuli (Fig. 1 F). They carried out 24–75 trials in 22–72 min in the last experiments. They were rewarded in 83–92% (85±4%, mean ± S.D.) of trials. Wilcoxon rank-sum tests showed that there were no significant differences between the unilateral and bilateral groups in the number of trials (p = 0.59), the duration (p = 0.22), the number of rewarded trials (p = 0.84), or the reaction time from the go-signal (p = 0.9), although the difference in the ratio of rewarded trials was marginally significant (p = 0.06).

### c-Fos-positive cell density maps

Figure 3 shows c-Fos-positive cell density maps (PCDMs) at five levels for each of the three task groups. The PCDMs of the test group (left column) are characterized by strong c-Fos expression in the superficial part of the dorsal cerebral cortices that are distributed over the primary and secondary motor cortices (M1 and M2 at Bregma +2, +1, and 0 mm from the bregma), the primary and secondary somatosensory cortices (S1, S1BF, and S2 at Bregma 0, −1, and −2 mm), and laterally over the secondary auditory cortices (AuD and AuV at Bregma −2 mm). On the other hand, this dorsal and superficial c-Fos expression was observed less readily in the unilateral group and barely in the home-cage group. Activation of the hypothalamus was commonly found in all three groups. High c-Fos-positive cell densities were found in the right amygdala in the unilateral stimulation group and the home-cage control group but less so in the test group (amygdala at Bregma −1 mm).

### c-Fos statistical parametric mapping

The statistical parametric mapping in Fig. 4 shows the differences among the groups in more detail. As shown in the right column, the unilateral group yielded significantly higher c-Fos-positive cell densities than the home-cage group in the barrel fields of the primary sensory cortex (S1BF at Bregma 0 and −1 mm), S1, M1, and M2 (Bregma 0 mm), the retrosplenial agranular and granular cortices (RSA and RSG, Bregma −1 mm), and the left striatum and left lateral hypothalamus (Bregma 0 mm).

The left column (Fig. 4) shows areas in which c-Fos-positive cell densities were significantly higher in the bilateral stimulation group than in the home-cage and unilateral stimulation groups. Some, if not all, of these areas are most likely related to ordering bilateral tactile signals in time. We found significantly higher expression (p<0.05, uncorrected) in the superficial layers of the bilateral barrel fields of S1 (S1BF at Bregma 0 and −1 mm), the left secondary somatosensory cortex (S2 at 0 and −1 mm), the dorsal part of the right secondary auditory cortex (AuD, −2 mm), the bilateral primary motor cortex (M1 at Bregma +1 and 0 mm), the right secondary motor cortex (M2 at Bregma +2, +1, and 0 mm), the right retrosplenial granular cortex (RSG at Bregma −1 mm), and the bilateral olfactory nuclei (Pir, Bregma +2 mm) in the bilateral test group.

### Single-labeling for c-Fos: cell counts in each layer

In the PCDM analysis (Fig. 3), both the unilateral and bilateral stimulation groups yielded c-Fos expression in the barrel fields of the primary somatosensory cortex. However, the expression of the test group was prominent in the superficial layers, whereas that of the unilateral group was mainly found in the deeper layers. These observations were generally supported by the c-Fos SPM analysis (Fig. 4), in which the test group showed significantly higher c-Fos expression in the superficial layers of the barrel cortex. To further verify this finding, we calculated the c-Fos-positive cell densities in each layer after identifying the supra- and infragranular layers with cytochrome oxidase staining (Fig. 5).

As exemplified in Fig. 5A and shown quantitatively in Fig. 5B, the c-Fos-positive cell density of the test group was the greatest of the three groups and was located in supragranular layers II and III in particular. The supragranular c-Fos-positive cell densities of the test group were significantly greater than those of layers II-III and IV of the unilateral stimulation group and those of all layers of the homecage group after a Bonferroni correction for multiple comparisons (α = 0.05, asterisks in Fig. 5B).

### Double-labeling

**Figure 6:**
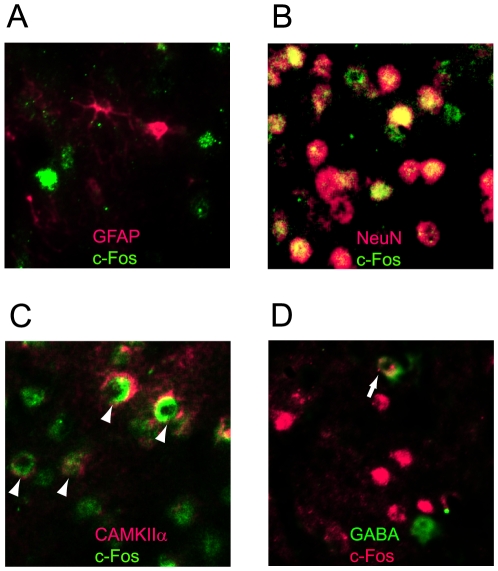
Double-staining with c-Fos and other antibodies. Images were taken from the barrel field of the mice in the test group. (A) c-Fos (green) and GFAP (red). No co-localization was observed. (B) c-Fos (green) and NeuN (red). Most cells were double-stained (yellow). (C) c-Fos (green) and CAMKIIα (red). Some cells were double-stained (arrow heads). (D) c-Fos (red) and GABA (green). Some cells were double-stained (arrows).

We further investigated the cellular types of the c-Fos-immunoreactive cells using double-labeling methods. We found that c-Fos never co-localized with GFAP, an astroglial marker (Fig. 6A), but generally (98%) co-localized with NeuN, a neuronal marker (Fig. 6B). We found that 26±4% (mean±s.e.m, 31 sections) of c-Fos positive cells were double-stained with CAMKIIα (e.g. Fig. 6A), a marker of excitatory neurons [37], while 21±4% (mean±s.e.m, 42 sections) were labeled with GABA (Fig. 6D). These results indicate that c-Fos positive cells in the test group were not glial cells but neuronal cells and that they were mixtures of excitatory neurons and inhibitory neurons.

## Discussion

In the present study, we applied statistical parametric mapping of immunopositive cell density [35], [38], [39] to c-Fos immuno-stained sections and illustrated differences in brain activity among three task groups. A critical comparison was made between the mice that judged the temporal order of successive stimuli to bilateral whiskers (test group) and those that oriented to the side of stimulation (unilateral group) without having to pay any attention to the temporal order. We found that c-Fos expression was significantly higher in the test group than in the unilateral group in several areas of the brain, including the superficial layers of the bilateral barrel cortex and the secondary sensory cortices. Given that the numbers of stimuli, responses, and rewarded trials and the reaction time from the go-signal were comparable between the two groups, we suggest that the aforementioned areas are critically involved in ordering successive somatosensory stimuli in time. However, it may be argued that the significantly higher c-Fos expression in the test group was caused by the difference in the age at sacrifice, 8 m.o. in the test group and 5 m.o. in the unilateral group (p = 0.048), and not by the difference in the tasks. This is not likely because c-Fos expression generally decreases with age [40], [41], [42], [43] and the test group was older than the unilateral group on average.

### c-Fos expression common to all three groups

c-Fos expression was commonly found in the hypothalamus. The expression in the hypothalamus might reflect stress or appetitive activities owing to the food restriction imposed on the three groups [44], [45], [46], [47], [48], [49]. We confirmed in one mouse that c-Fos expression in the paraventricular hypothalamic nucleus disappeared when there was no food restriction [50].

### c-Fos expression specific to the test group

c-Fos expression was significantly higher in the test group than in the unilateral group in the following areas: the superficial layers of the bilateral barrel fields of the primary somatosensory cortex (S1BF), left secondary somatosensory cortex (S2), dorsal part of the right secondary auditory cortex (AuD), bilateral primary motor cortex (M1), right secondary motor cortex (M2), right retrosplenial granular cortex (RSG), and bilateral olfactory nuclei.

In the following discussion, we detail on an individual basis whether and how these areas might contribute to ordering bilateral sensory stimuli in time.

#### Supragranular layers in the barrel cortex

c-Fos statistical parametric mapping and more detailed laminar analyses revealed that the c-Fos-positive cell density was significantly greater in supragranular layers II and III of the barrel cortex in the test group than in the other control groups (Fig. 5B). The expression in the supragranular layers was in marked contrast to previous studies that reported c-Fos expression in the granular layer (layer IV) in response to tactile stimulation to whiskers [23], [25], [26], [36]. While the granular layer receives unilateral somatosensory signals from the thalamus, the supragranular layers send and receive callosal fibers that transfer information from one hemisphere to the other [51], [52], [53]. Shuler et al.[54] showed that signals from the right and left whiskers converge on single neurons of the primary somatosensory cortex in rats. In these neurons, stimuli to the contralateral whiskers evoke neural activities with a sharp onset and short duration that can be distinguished from those evoked by stimulation to the ipsilateral whiskers with an indistinct onset and longer duration [54]. Thus, the temporal organization of discharge in single neurons in the barrel field encodes sufficient information for judging temporal order. The particular expression of c-Fos in the superficial layers suggests that convergent signals from bilateral whiskers in the barrel field were actually utilized in ordering bilateral stimuli to the whiskers.

#### Secondary sensory cortices

Even if the temporal order of bilateral stimuli is encoded in single neurons in the barrel field, it does not mean that the information is decoded in the barrel field. In this context, it is worth noting that c-Fos expression in the test group was significantly higher in the secondary somatosensory cortex (S2) and the dorsal part of the secondary auditory cortex (A2), both of which are located near the border of the parietal (somatosensory) and temporal (auditory) cortices. Recent electrophysiological studies in rats [55], [56] have shown that there is a distinct multisensory (both somatosensory and auditory) area near the border of the two sensory modalities. Thus, the c-Fos expression in these secondary sensory cortices suggests that multimodal neurons are involved in judging the temporal order of stimuli that are unimodal (somatosensory) in origin. It may be argued, however, that the involvement of the secondary auditory cortex is an artifact attributable to the sounds generated by the air-puff stimuli. We may be able to eliminate this possibility for the following reasons. First, the corrected response rates dropped nearly to the level of chance when the whiskers were removed under the same experimental paradigm [20], indicating that the mice in the test group depended on somatosensory but not auditory signals for their judgment. Second, both the test and control groups received the same white noises, and there were indeed no significant differences in the c-Fos expression in the primary auditory cortex. This suggests that the origin of the difference in the secondary auditory cortex was not auditory but was instead somatosensory.

The suggested involvement of multisensory areas in ordering somatosensory signals in time agrees in part with our previous findings in human subjects [9], [10], [11], [12]. In these studies, we showed that human subjects were not basing the judgment of temporal order of two tactile stimuli on the somatotopical map but rather on a spatial map. Therefore, it is intriguing to consider whether multisensory areas in the rodent cerebral cortex represent a spatial map that is shared by multiple sensory modalities.

#### Motor cortices

We found significantly stronger c-Fos expression in the motor cortices. The expression was most evident in the secondary motor cortex, which lies rostrally to the barrel cortex and extends over the most medial part of the cerebral cortex [31]. According to recent studies in rats[57], [58], the medial part of the cerebral cortex receives abundant cortico-cortical projections from the barrel cortex, and microstimulation to the area evokes low-threshold movements of the vibrissae. Thus, we suggest that the area of c-Fos expression in the present study corresponds to the vibrissal representation of the primary motor cortex, although the area in mice has been labeled as the secondary motor cortex [31]. From our results, we raise the possibility that whisker movements are optimally controlled in the test group so that temporal differences of bilateral air-puff stimuli can be efficiently detected.

#### Other areas

c-Fos expression in the olfactory nuclei was also enhanced in the test group. The reason for this activation is not clear at present, but we hypothesize that some pattern of whisker movements specific to the test group, such as sniffing, might have enhanced the sensitivity to olfaction. To verify this hypothesis, actual patterns of whisker movements should be examined in each task group.

The retrosplenial granular cortex was another area of c-Fos expression in the test group. This area has been known to be important for a T-maze task in mice [59], but the relevance to the temporal order judgment task remains to be elucidated.

### Implications for the mechanisms of temporal order judgment

Dennet and Kinsbourne [13] claimed that, in judging temporal order of sensory signals, it is advantageous to encode the order of the signals near the entrance of these signals before the physical order is lost. The encoded signal can then be stably sent elsewhere to effectively use the information. From the results in the present study, we hypothesize that the temporal order of bilateral whisker stimuli is encoded in the neural activity in the supragranular layers of the barrel cortex. We do not know at present whether the adjacent secondary sensory cortices also contribute to encoding the order of the signals or use the encoded temporal order for further judgment. The present study has provided solid clues as to where the neural codes for temporal order should be sought.
